# New Appliances and Things Medical

**Published:** 1896-12-12

**Authors:** 


### NEW APPLIANCES AND THINGS MEDICAL.

[We shall be glad to receive, at our Office, 28 & 29, Southampton Street, Strand, London, W.C., from the manufacturers, specimens of all new preparations and appliances which may be brought out from time to time.]

### PURE LIQUORICE JUICE.

(Fergusson and Forster, Great Tower Street, E.C.)

We have received a sample of the well-known Solazzi liquorice. It is prepared in large sticks, and from trial which we have made of it it may be regarded as a remarkably pure sample. From its complete solubility and freedom from adulteration it may be safely relied upon as a therapeutic agent of great value.

### MEDICAL SOAPS.

(Allen and Hanburys, Plough Court, Lombard Street, E.C.)

We have examined with considerable interest some samples of medicated soaps which have been forwarded us by the above firm, and we are glad to learn that an English firm of so high a reputation is undertaking the manufacture of these valuable adjuncts to dermatological treatment, for up to the present they have been almost the exclusive monopoly of German houses. The common basis of all these soaps appears to be of first-rate quality, of nearly neutral reaction, and affording a soft, agreeable lather. The milling, medication and compression is carried out by Messrs. Allen and Hanbury, a sufficient guarantee of correct standardisation and medication with drugs of the best quality. The list includes soaps containing the following substances: Beech tar, benzoin, boracic, camphor and menthol, camphor and balsam of Peru, carbolic acid, coal tar, ichthyol, Juniper tar, and this list will doubtless be largely extended, as demands from the profession make this step inevitable.

### FILMOGEN.

(Thomas Christy and Co., 25, Lime Street, London, E.C.)

This is a new medium for applying to the skin many of the drugs which are most useful in dermatological practice. When painted on the skin it rapidly forms a thin, dry, flexible film, which adheres firmly and is not removed by the ordinary process of washing. It has distinct advantages over the various forms of varnish, collodion, and gelatine jelly at present so largely in use. Among the medicated varieties of filmogen supplied by Messrs. Christy may be mentioned salicylic acid, resorsin, rodoform, pyrogallol, cocaine, ichthyol, these and many others being soluble in this new medium, which consists of a solution of nitrated cellulose, acitone, and a small quanity of oil; such substances as sulphur, oxide of zinc, lead iodide, &c., which are insoluble in the medium, are easily held in suspension and applied to the skin in the form of an intimate mixture. Filmogen, which is Dr. Schiff's invention, is certainly a great addition to the resources of artistic surgery.

